# Effect of Enhanced Squeezing Needle Structure on the Jetting Performance of a Piezostack-Driven Dispenser

**DOI:** 10.3390/mi10120850

**Published:** 2019-12-05

**Authors:** Xiang Huang, Xiaolong Lin, Hang Jin, Siying Lin, Zhenxiang Bu, Gonghan He, Daoheng Sun, Lingyun Wang

**Affiliations:** 1 Department of Mechanical and Electrical Engineering, Xiamen University, 4221-134th Xiang’an South Road, Xiamen 361102, Chinalinxiaolong@stu.xmu.edu.cn (X.L.); jinhang@stu.xmu.edu.cn (H.J.); linsiying1226@stu.xmu.edu.cn (S.L.); buzhenxiang108@stu.xmu.edu.cn (Z.B.); hgh@xmu.edu.cn (G.H.); sundh@xmu.edu.cn (D.S.); 2 Shenzhen Research Institute of Xiamen University, 19th Gaoxin South Fourth Road, Shenzhen 518000, China

**Keywords:** needle structure influence, piezostack-driven dispenser, dispensing process, droplet variation range

## Abstract

Advanced dispensing technology is urgently needed to improve the jetting performance of fluid to meet the requirements of electronic product integration and miniaturization. In this work, an on–off valve piezostack-driven dispenser was used as a study object to investigate the effect of needle structure on jetting performance. Based on fluid dynamics, we investigated nozzle cavity pressure and jet velocity during the dispensing process using theoretical simulation for needles with and without a side cap. The results showed that the needle with a side cap had larger jet velocity and was capable of generating 8.27 MPa of pressure in the nozzle cavity, which was 2.39 times larger than the needle without a side cap. Further research on the influence of the nozzle and needle structural parameters showed that a nozzle conic angle of 85°–105°, needle conic angle of 10°–35°, and side clearance of 0.1–0.3 mm produced a dispenser with a large jet velocity and stable performance, capable of dispensing microscale droplets. Finally, a smaller droplet diameter of 0.42 mm was achieved in experiments using a glycerol/ethanol mixture, with a variation range of ± 4.61%.

## 1. Introduction

Fluid dispensing technology controls the quantitative distribution of liquid to specific areas in a device [1,2,3]. Nowadays, dispensing systems are widely used in many fields and industries, including in the photovoltaic solar energy industry, medical applications, light emitting diode (LED) technology and the electronic component packaging industry [4,5,6]. According to the dispensing principle, dispensing systems can be divided into contact dispensers and jetting dispensers. The needle of contact dispensers needs to be in contact with the substrate, which means it is easy to damage the device. In addition, contact dispensers have low efficiency, and since the dispenser squeezes the fluid by applying air pressure, piston movement or screw rotation, the size accuracy of droplets is difficult to control and therefore challenging for use in the packaging of micro-devices in Micro Electro Mechanical Systems (MEMS) and other fields [7,8,9].

Jetting dispensers effectively overcome the shortcomings of contact dispensers. These dispensers instantaneously create high pressure in the nozzle cavity to squeeze liquid out. Movement of the vertical substrate direction is avoided, greatly improving the working efficiency and protecting the needle [10,11,12,13]. In particular, piezostack-driven dispensers are driven by the displacement of a piezoelectric actuator. In such devices, the needle can obtain large movement velocity thanks to the quick response of the piezoelectric actuator. This produces a large shear force that can overcome the viscous forces of the jet, breaking it. Moreover, piezostack-driven dispensers have various applicable fluids, good droplet consistency and high frequency operating characteristics [14,15,16,17]. Therefore, research on piezostack-driven dispensing systems has aroused enormous excitement, particularly research on the displacement amplifying mechanism and jetting mechanism [18,19]. Reasonable structure to the jetting mechanism is an important factor in the performance of such dispensers, and involves careful design of the flow channel, nozzle and needle. Zhou et al. [20] studied the influence of flow channel structure through simulations and experimentation. Their results showed that, as the channel width increased, the flow velocity and pressure decreased, while the droplet volume increased. Lu et al. [21] performed a study to improve the viscosity of an applied fluid through changing the nozzle spray glue striker structure. They found that, as the needle radius was increased, the instantaneous pressure generated in the injection chamber increased (along with jet velocity), but the nozzle taper angle and diameter decreased. Moreover, they found overcoming the viscous force of the fluid easy. In the past, some of the authors of the present study developed simulations and experiments to study the influence of nozzle diameter on flow behavior [22]. Larger nozzle diameters were found to lead to fluid with more kinetic energy in the nozzle, and so the maximum velocity of the fluid increased.

This study mainly focused on the influence of needle structure on an on–off valve piezostack-driven dispenser’s jetting process. A displacement amplifier with two piezoelectric actuators was used, and its working principle is introduced herein. The effect of needles with and without a side cap on nozzle cavity pressure was then investigated and fluid velocities from the nozzle outlet during dispensing were analyzed by simulation. Moreover, parameters of the needle and nozzle were determined and processed for experiments. Finally, a control method for the dispenser with an enhanced squeezing needle was explored.

## 2. Principles of Dispensing

**Figure 1:**
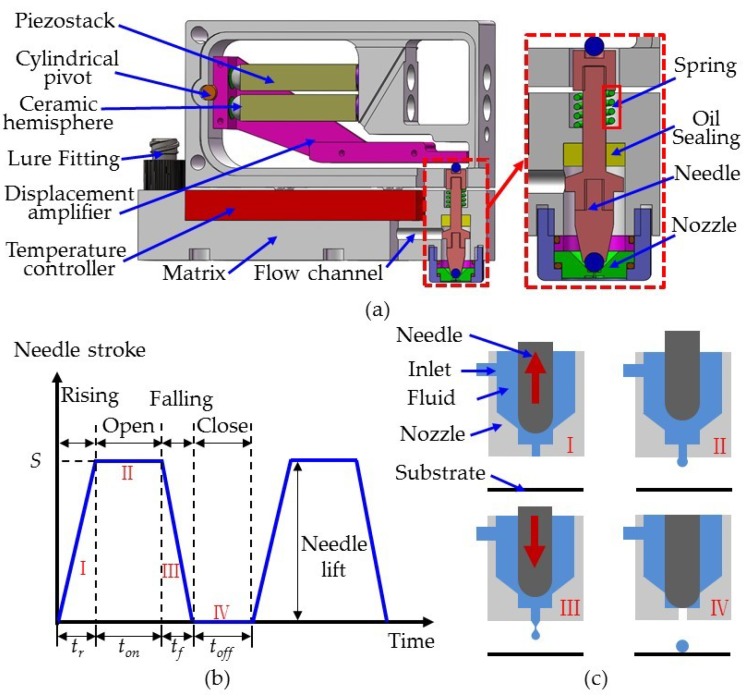
Configuration of the jetting dispenser and its dispensing process: (**a**) structure of piezostack-driven dispenser; (**b**) needle stroke curve during dispensing; and (**c**) nozzle status at different stages.

In order to study the influence of needle structure on the dispensing process, an on–off valve piezostack-driven dispenser was used, the configuration of which is shown in Figure 1a. Figure 1a also shows the displacement amplification and jetting mechanisms. For the displacement amplification mechanism, a cylindrical pivot was connected to a matrix and displacement amplifier. Two piezostack actuators with ceramic hemispheres were symmetrically distributed at both ends of the lever arm of the displacement amplifier. The jetting mechanism was separated from the displacement amplification mechanism, and was composed of a nozzle, needle, flow channel, temperature controller, Luer fitter, and spring, as well as an oil seal. The Luer fitter was connected to a cylinder containing various fluids, which entered the nozzle cavity through the flow channel. Under the action of the spring, the needle remained in contact with the end of the displacement amplifier. When the upper and lower piezostack actuators alternately elongated during dispensing, the displacement amplifier rotated a small angle, causing reciprocating movement in the needle. Figure 1b,c shows the jetting process of the dispenser. When the dispenser was energized, the lower piezostack actuator elongated, the upper piezostack actuator maintained its original length, and the displacement amplifier rotated a small angle clockwise, driving the needle until it reached the lowest position. At this time, the needle stroke was considered zero. According to the different phases of the needle and nozzle in the dispensing process, four stages were recognized, namely stage I, II, III and IV. In stage I, the upper piezostack actuator elongated, the lower piezostack actuator resumed its original length, and the needle rose. At this point, fluid entered the gap between the nozzle and needle under feed pressure. During stage II, the upper piezostack actuator reached its maximum elongation, the lower piezostack actuator maintained its original length, and the needle reached its highest position. Here, the nozzle opened and the fluid flowed out from the nozzle outlet. In stage III, the upper piezostack actuator returned to its original length and the lower piezostack actuator extended. Then, the needle dropped rapidly, producing a large shear force that overcame the viscous forces of the fluid, breaking the jet. Finally, at stage IV, the nozzle closed, and the fluid formed a round droplet on the substrate. Stages I–IV form a complete cycle of dispensing, with durations t_r_, t_on_, t_f_ and t_off_, respectively. Therefore, dispensing cycle T = t_r_ + t_on_ + t_f_ + t_off_.

By analyzing the jetting process of the dispenser, smooth dispensing of fluid should meet the following conditions. (1) Reasonable feed pressure. Although sufficiently high pressure can ensure smooth jetting of the fluid, it has a great influence on the shape of the droplet. For a fluid with small surface tension, high feed pressure will form a larger droplet, which is a contamination of the packaged devices. (2) Suitable nozzle and needle matching form. The matching between a needle and nozzle usually involves linear contact, and so suitable matching form can ensure the fluid has sufficient kinetic energy during dispensing. In order to completely cut off a fluid, it is necessary to study the influence of needle–nozzle matching form on pressure and velocity. (3) Strong squeezing capacity of the needle. Effective needle structure can provide greater squeezing pressure in the small gap, thereby accumulating kinetic energy for jetting of the fluid. However, an improvement of squeezing capacity also means that there will be appreciable suction as the needle rises, so a slight increase of supply pressure is needed to make up for it.

## 3. Theory and Simulation of the Dispensing Process

### 3.1. Theoretical Analysis

**Figure 2:**
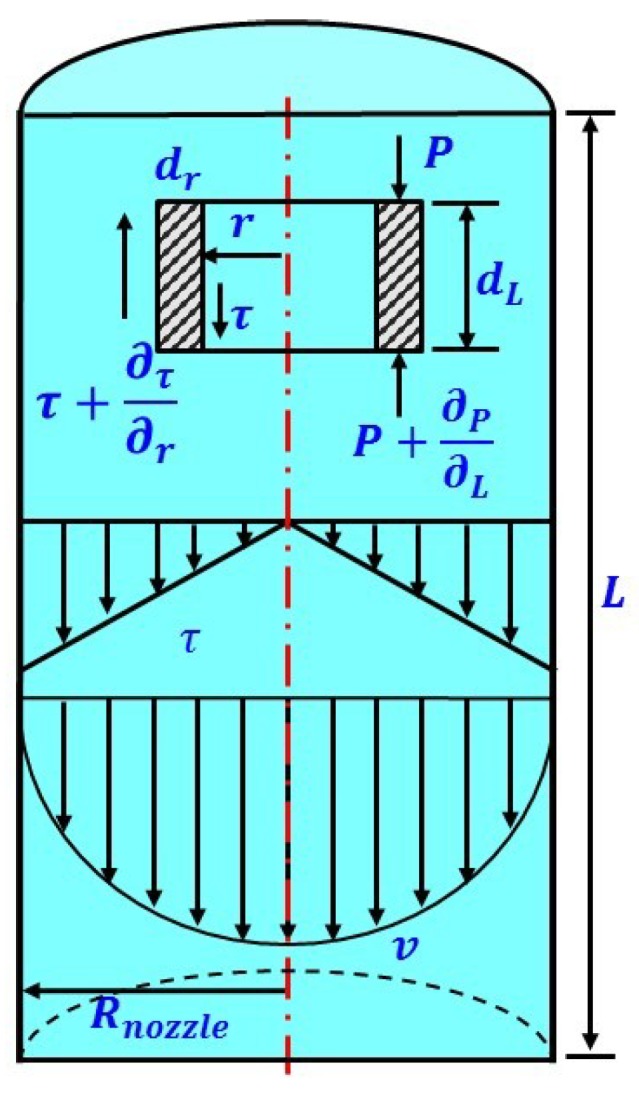
Schematic diagram of fluid dynamics in the nozzle pipe.

Study of the dispensing process required knowledge of the fluid’s flow characteristics in the nozzle pipe, which were affected by the characteristics of the liquid, structure of the dispensing mechanism and characteristics of fluid flow. Flow characteristics include fluid properties such as viscosity, density, surface tension, and compressibility, and structural parameters such as nozzle diameter, nozzle length, and boundary deformation, as well as the effects of differential pressure of the nozzle inlet and outlet, gravity, friction, acceleration, boundary slip and continuity of fluid flow. However, some factors have little influence and can be ignored or approximated, which is beneficial for simplifying a model. In our simplified model, the following assumptions were made. (1) The fluid is incompressible and the flow of the fluid during jet formation satisfies the continuity principle. That is, the mass of the fluid flowing into any section should be equal to the mass of the fluid flowing out of any other section at the same time. (2) The effect of gravity is negligible. (3) The effect of shear force on fluid viscosity is ignored. (4) The fluid wall has no deformation and no slip boundary. (5) The axial flow velocity is much higher than the radial flow velocity, so that the radial flow can be ignored. Figure 2 is a schematic diagram of the fluid dynamics in the nozzle pipe, with the circular micro-element used for analysis under cylindrical coordinates. In it, the element has a radius of r, a thickness of d_r_, and a length of d_L_, while the shear force is τ and the pressure force on the end surface is p. According to the no-slip boundary condition, the velocity of a power-law fluid in the nozzle is as follows [23,24]:

(1) $$v(r)=\frac{n}{n+1}{\left(\frac{\Delta P}{2\eta L}\right)}^{1/n}\left(R_{nozzle}^{{}^{1+1/n}}-r^{1+1/n}\right)$$

where R_nozzle_ is the radius of the nozzle, ∆P is the pressure difference between the inside and outside of the nozzle, L is the length of the nozzle tube, η is the dynamic viscosity, and n is the flow exponent. The flow exponent n, also known as the non-Newtonian exponent, represents the degree of deviation between the fluid and a Newtonian fluid. Specifically, n = 1 corresponds to a Newtonian fluid, n < 1 corresponds to a dilatant fluid, and n > 1 corresponds to a pseudoplastic fluid. The velocity of the fluid along the central axis of the nozzle, v_c_, is the maximum; that is, when r = 0,

(2) $$v_{c}=\frac{n}{n+1}{\left(\frac{\Delta P}{2\eta L}\right)}^{1/n}R_{nozzle}^{{}^{1+1/n}}$$

Actually, the movement of the needle makes fluid flow unsteady in the nozzle,

(3) $$v(t,r)=v_{c}(t)[1-{\left(\frac{r}{R_{nozzle}}\right)}^{1+1/n}]$$

The liquid flow Q and dispensing volume V of a cycle are respectively expressed as:

(4) $$Q=\int_{0}^{R_{nozzle}}2\pi rv(t,r)dr=\frac{n+1}{3n+1}\pi R_{nozzle}^{2}v_{c}(t)$$

(5) $$V=\int_{0}^{T}Qdt=\frac{n+1}{3n+1}\pi R_{nozzle}^{2}\int_{0}^{T}v_{c}(t)dt$$

where T is the time for a cycle. According to the above results, the dispenser’s dispensing volume is not only affected by the nozzle diameter and properties of the fluid, but also by the velocity of the fluid and pressure inside the nozzle.

### 3.2. Molding and Simulation

**Figure 3:**
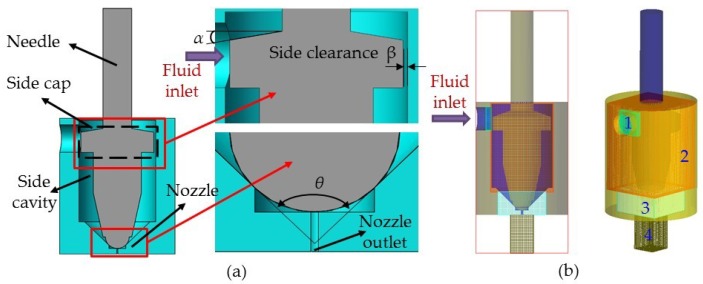
Simulation model of the needle and nozzle. (**a**) Parameter dimensions of the simulated mold and (**b**) mesh generation.

For the purpose of obtaining the universal law, a fluid dynamics simulation of the dispensing process was carried out, and the influence of each parameter on both the velocity of the fluid and volume of the droplet was analyzed. In this study, a needle with a side cap was designed to increase the pressure in the nozzle cavity and the velocity of the jet, so that the fluid was able to flow out more easily. The side cap was also expected to have a particular role in inhibiting reflux. Moreover, a spherical–conical combination of needle and nozzle was selected, which had good sealing performance. Figure 3a shows the parameter dimensions of the simulated mold. The initial parameters of the model were set as follows: conic angle α of needle was 20°, conic angle θ of nozzle was 90°, and side clearance β was 0.1 mm. In addition, the length of the nozzle pipe was maintained at 1 mm, the aperture of the nozzle at 0.15 mm, and the diameter of the needle head at 2.5 mm. Mesh generation is shown in Figure 3b. As the structure was not symmetric about the central axis, the three-dimensional model was adopted for simulation. The whole structure was divided into four grid blocks to save computing resources. Among them, mesh 3, being at the contact site between the needle and the nozzle, contained fine structures, so that the mesh needed to be refined.

**Figure 4:**
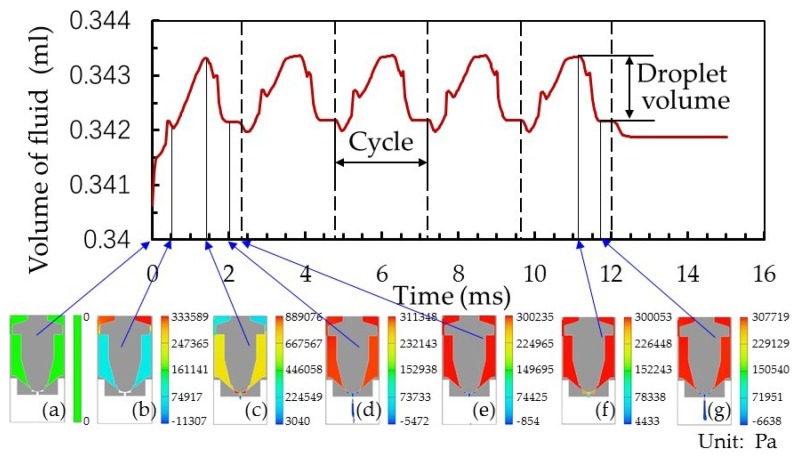
Fluid volume changes in the nozzle cavity. (**a**) The beginning of the dispensing process; (**b**) the needle reaches its highest position; (**c**) the needle starts to fall; (**d**) the needle strikes the nozzle; (**e**) the end of the first dispensing cycle; (**f**) the fluid begins to flow out of the nozzle; and (**g**) the jet flow is cut off in the fifth cycle.

The initial values of each parameter for the fluid dynamics simulation are shown in Table 1. At the starting point, the nozzle cavity was filled with fluid with the nozzle closed, and there was no fluid in the nozzle pipe. By analyzing the volume of fluid change in the nozzle cavity (see Figure 4), the dispensing process was divided into four stages: fluid filling, jet flow forming, jet drops and droplet deposition. During the dispensing process, the rise and fall of the needle generated overflow space, squeezing the fluid so that its volume in the nozzle cavity changed. These pressure changes and jet flow formation at different needle positions over a specific period are shown in Figure 4. We defined droplet volume as the change of fluid volume in the nozzle cavity over the period in which the needle initially fell until it came into contact with the nozzle (Figure 4f,g).

**Figure 5:**
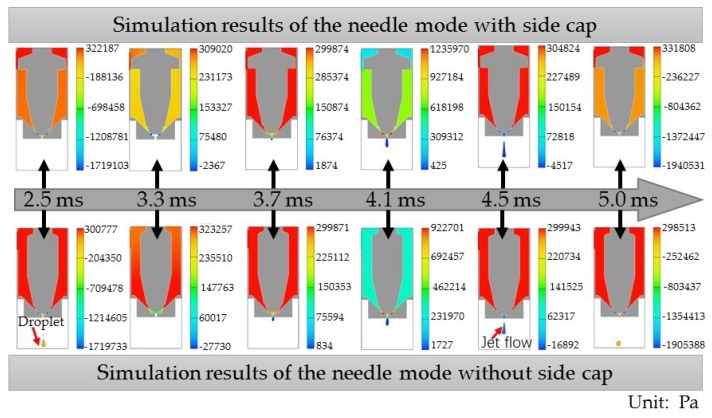
The simulation of dispensing processes with different needle models for pressure in the nozzle cavity and a jet velocity comparison.

While keeping the other variables consistent, a needle without a side cap was employed for comparison. The dispensing processes of the second cycle are shown in Figure 5. It can be seen from the results that the needle model with a side cap had an enhanced squeezing effect on the fluid, with the jet flow possessing a higher jetting velocity. At 2.5 ms, the droplet of the previous period was still within the boundary of the needle model without a cap, while the droplet had flown out of the boundary in the simulation of the needle model without a cap. At 3.3 ms, the fluid filled the step groove more quickly in the case of the needle model without a side cap. Because the gap between its nozzle seat and needle was larger, the feed pressure’s squeezing effect on the fluid was more apparent, and therefore squeezed more fluid out of the nozzle outlet. At 3.7 ms, pressure in the nozzle cavity of the needle models with and without a side cap were essentially equal. At 4.1 ms, pressure in the nozzle cavity of the needle model with a side cap was greater than that of the needle model without a side cap. During the falling process of the needle, the needle model with a side cap inhibited fluid reflux in the cavity, providing more energy to the jet. At 4.5 ms, the jet flows produced by both models were broken. Moreover, the jet velocity of the needle model with a side cap was greater than the model without a side cap, which can be observed by comparing the results obtained at 5.0 ms.

**Figure 6:**
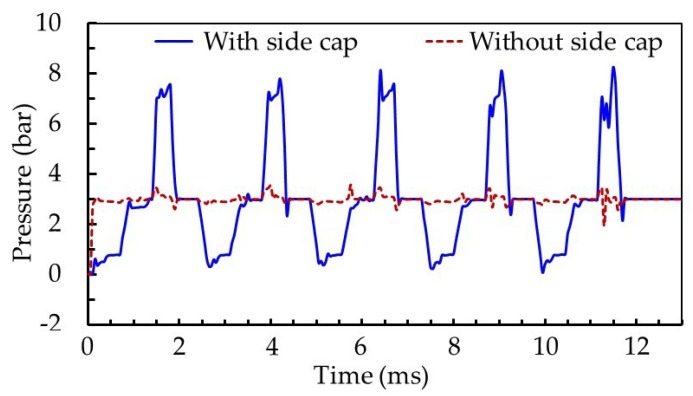
Pressure variation in the cavity of the models with and without side caps by simulations.

Figure 6 shows pressure variation in the side cavity of the molds with and without side caps, obtained by simulations. Compared with the needle model without a side cap, the pressure of the nozzle side cavity changed significantly during the rising and falling process of the needle with a side cap. The maximum pressure of the nozzle cavity in a single cycle was 8.27 MPa, which was about 2.39 times that of the model without a side cap. The parameter of volume change ratio in the nozzle cavity was used to evaluate the degree of fluid compression in the nozzle cavity, and is the ratio of fluid filling amount to nozzle cavity volume in one cycle. For the needle with a side cap, the ratio of volume change in nozzle cavity was 0.341%, which was greater than that of the needle without a side cap (0.315%). A large volume change rate generated greater volume compression of the fluid, which made it easier to obtain high pressure in the nozzle cavity. Moreover, the gap between the needle with a side cap and the inner wall of the nozzle cavity was smaller. When the needle was at its lowest position, the small gap was not conducive to fluid entering the cavity below the side cap, resulting in a large pressure difference between the upper and lower cavities separated by the side cap. This corresponds to the simulation results of images at 2.5 ms in Figure 5. As the needle rose, fluid was forced through the inlet into the lower cavity. Also, a small gap was beneficial to maintaining pressure. Pressure in the nozzle cavity was greatest with the needle in its highest position (images at 4.1 ms in Figure 5). As the needle dropped, pressure decreased sharply, and the fluid obtained more kinetic energy. Under the action of shear forces produced by the needle, the jet broke and had a large velocity, as reflected in Figure 5 in images at 5.0 ms. Therefore, the needle with a side cap produced a jet flow with large velocity, which is conducive to the dispenser’s high frequency operation. However, the side cap makes it difficult for fluid to fill the cavity. Thus, a reasonable gap between the nozzle and needle is needed to ensure smooth fluid supply. 

The droplet volume produced by the needles with and without side caps was also assessed, as shown in Table 2. The average diameter of the droplets produced by the needle with a side cap in five cycles was 1.166 μL, which was smaller than that produced by the needle without a side cap (1.234 μL). The gap between the nozzle and the needle without a side cover was larger and the fluid resistance was smaller, meaning the amount of fluid flowing into the nozzle per unit time was larger. In addition, the needle with a side cap was able to generate higher pressure and obtain large shear forces, so that the jet was cut off in time.

**Figure 7:**
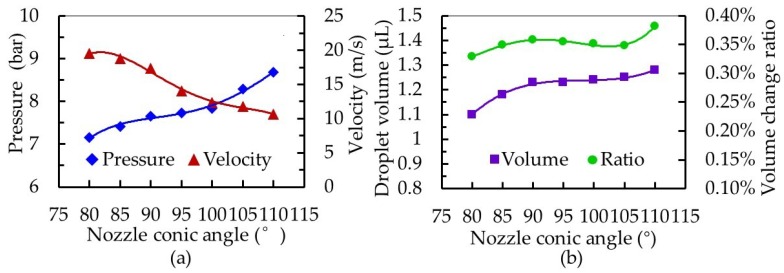
The influence of nozzle conic angle on the dispensing process. (**a**) Effect on the pressure in the cavity and velocity at the nozzle outlet and (**b**) effect on the droplet volume and volume change ratio in the nozzle cavity.

The effects of model structural parameters on the dispensing process were studied in order to design an enhanced squeeze needle and nozzle suitable for microscale droplet distribution. Figure 7 shows the influence of the nozzle conic angle on the dispensing process. As the nozzle cone angle increased from 80° to 110°, the pressure in the nozzle cavity increased from 7.15 to 8.68 bar, while the jet velocity at the nozzle outlet decreased from 19.54 to 10.60 m/s. Moreover, the droplet volume changed from 1.10 to 1.28 μL, and the volume change ratio in the nozzle cavity increased from 0.329% to 0.382%. In this study, the spherical–conical combination of needle and nozzle was selected, which were sealed by line contact. An increase of the nozzle’s cone angle caused the space formed after contact between the nozzle and needle to become smaller, which caused the ratio of volume change to become larger, and was conducive for generation of larger pressure. However, the gap above the contact line increased, resulting in serious backflow of the fluid and a decrease in the jet velocity. In addition, when the needle stroke was constant, the distance between the contact line and the highest position of the needle’s movement became larger as the nozzle angle increased, meaning it took a relatively long time for the fluid to flow out of the nozzle in one cycle and an increase in the size of the droplet. Therefore, for dispensing tests, an appropriate nozzle conic angle is needed for different fluids.

**Figure 8:**
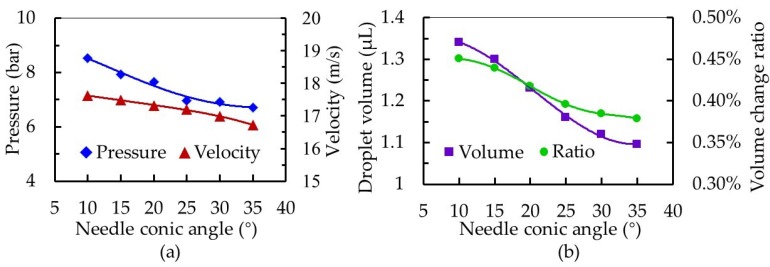
The influence of needle conic angle on the dispensing process. (**a**) Effect on the pressure in the cavity and the velocity at the nozzle outlet and (**b**) effect on the droplet volume and volume change ratio in the nozzle cavity.

In this design, the needle conical angle determined the strength of suction. The larger the angle, the easier the fluid filled the gap when the needle was lifted, and the weaker the back suction. Therefore, while other structural parameters and conditions were kept constant, we changed the needle conic angle. Results of the simulation are shown in Figure 8. As the needle conic angle increased from 10° to 35°, the pressure in the nozzle cavity decreased from 8.53 to 6.70 bar and jet velocity decreased from 17.62 to 16.93 m/s. Furthermore, droplet volume decreased from 1.34 to 1.10 μL and the volume change ratio in the nozzle cavity decreased from 0.451% to 0.379%. A large needle conic angle α weakened the back suction effect during fluid dispensing, but also significantly reduced the volume change ratio in the nozzle cavity and decreased the squeezing pressure, resulting in a slow rise of pressure in the cavity. This caused a decrease in velocity at the nozzle outlet, and reduced the volume of droplets. From these results, a needle with a large conic angle is suitable for microscale droplet distribution.

**Figure 9:**
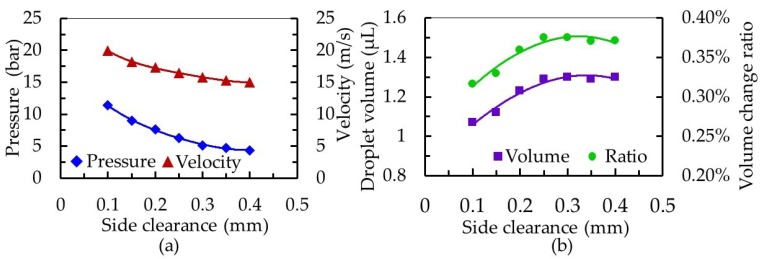
The influence of needle and nozzle matching side clearance on the dispensing process. (**a**) Effect on the pressure in the cavity and the velocity at the nozzle outlet and (**b**) effect on the droplet volume and volume change ratio in the nozzle cavity.

In the nozzle cavity, the value attributed to the side clearance between the needle and the inner wall of the nozzle affects the stability of the fluid supply, and the smaller the side clearance, the stronger the back suction and squeeze effect. From the results shown in Figure 9, as the side clearance increased from 0.1 to 0.4 mm, the pressure in the nozzle cavity decreased from 11.41 to 4.38 bar, and the jet velocity decreased from 19.95 to 14.98 m/s. Furthermore, this side clearance increase was associated with a droplet volume increase of 1.07 to 1.30 μL and an increase of the volume change ratio in the nozzle cavity, from 0.316% to 0.371%. When the side clearance increased to more than 0.25 mm, a reduction in the squeeze pressure made the advantage of a timely fluid supply no longer obvious, and the change of droplet volume tended to be stable. Although the volume change ratio in the nozzle cavity increased with an increase of the side gap, energy loss caused by fluid backflow was serious, leading to an increase in pressure in the nozzle cavity. In addition, the small clearance made jet velocity larger, but the resistance of fluid flowing through the clearance increased. When the needle moved, the amount of fluid in the overflow space per unit time was less than that of the large clearance. Therefore, on the basis of sufficient fluid supply of a dispenser, it is possible to obtain smaller droplets by controlling the working frequency of the dispenser. 

In summary, with the proper viscosity of the fluid, the parameters—pressure of 3 bar, nozzle conic angle θ of 85°–105°, needle conic angle α of 10°–35° and side clearance β of 0.1–0.3 mm—produced a dispenser with a large jet velocity and stable performance, capable of dispensing microscale droplets.

## 4. Experiments of Droplet Dispensing

**Figure 10:**
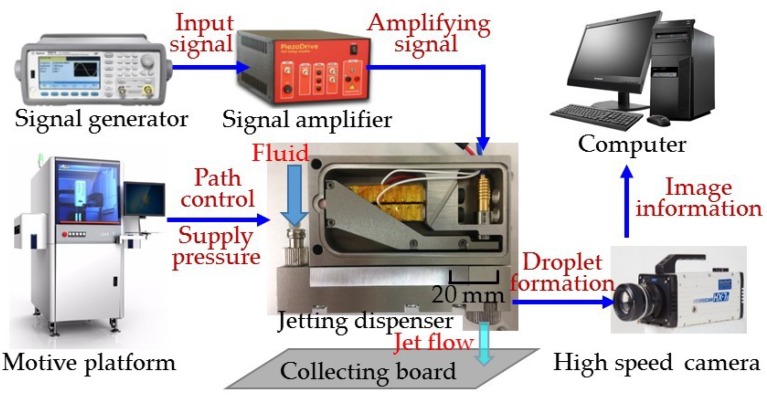
Experimental test system of the piezostack-driven dispenser.

The piezostack-driven dispenser experimental platform is shown in Figure 10. The experiment test system was composed of the jetting dispenser (81 mm × 62 mm × 15 mm), driven by double piezostack actuators, a motive platform, a high-speed camera, a computer, a collecting board, a signal generator and a signal amplifier. The motive platform was used to manage the dispensing path and adjust the feeding pressure. The high-speed camera was adopted to record the process of a droplet forming, with the captured images processed by the computer. A square wave signal was created by the signal generator, which was adjusted by the amplifier to excite the piezostack actuator. Needles with and without side caps were machined for testing, and the parameters of needle and nozzle were set as: a needle head diameter of 2.5 mm, nozzle aperture of 0.15 mm, nozzle pipe length of 1 mm and nozzle conic angle of 90°. For the needle with a side cap, a needle conic angle of 30° and a side clearance of 0.2 mm was selected. In the experiment, a glycerol/ethanol mixture with a viscosity of 500 mPa∙s was used. Also, the feed pressure was maintained at 0.3 MPa and the square wave voltage at 144 V.

**Figure 11:**
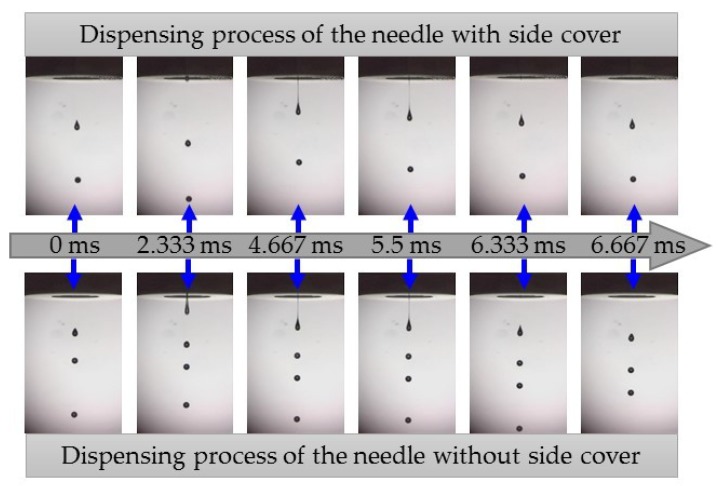
Droplet formation processes of the needle with and without a side cap.

The dispensing process was recorded using a high-speed digital camera, with some of the images captured presented in Figure 11. At a frequency of 150 Hz and a duty cycle of 20%, the results showed that the needle with a side cap enabled jet flow to fall faster than the needle without a side cap. The imaging interval of the high-speed camera was set to 6000 frames per second. Therefore, 40 images were able to be obtained in one allocation cycle with an interval of about 1/6000 s. Meanwhile, the last image of the previous cycle was considered the beginning of the next cycle. We selected some images for comparison. For the dispenser with the needle without a side cap, the fluid flowed out of the nozzle aperture earlier, but the breakup time of the jet was basically consistent with that of the needle with a side cap. In the dispensing process of the needle with a side cap, the number of droplets in the imaging field of the high-speed camera was fewer than that for the needle without side cap. This means that the needle with a side cap had an enhanced squeezing effect on the fluid, so that the falling velocity of the jet was large after breaking.

**Figure 12:**
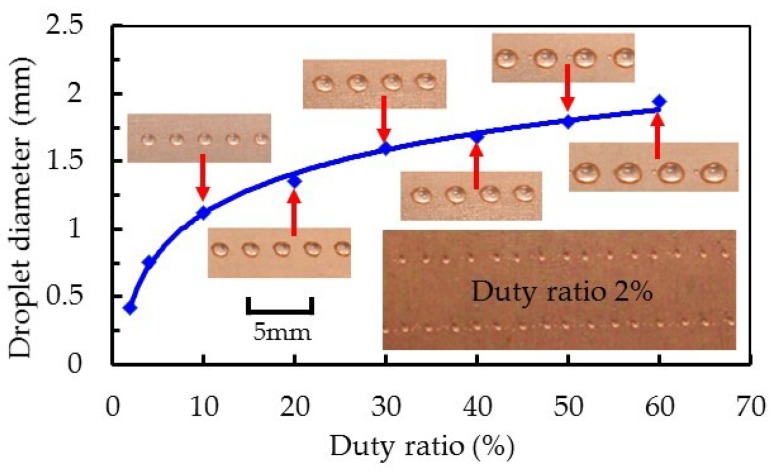
Relationship between square-wave duty cycle and droplet diameter (nozzle diameter: 200 µm; feed pressure: 3 bar; voltage amplitude: 144 V; frequency: 100 Hz; viscosity: 500 mPa∙s).

As we believed the working performance of the dispenser with an enhanced squeeze needle could be improved further tests were performed. Moreover, we determined a control method for the dispenser. Figure 12 shows the droplet diameter variation, obtained by changing the duty ratio of the square-wave signal when the operating frequency was 100 Hz. As the duty ratio changed from 2% to 60%, the diameter of the droplet increased from 0.42 to 1.94 mm, and the variation trend gradually leveled off. Note that the minimum droplet diameter was 0.42 mm, corresponding to a duty ratio of 2%. As the opening time of the nozzle increased, the flow rate of fluid out of the nozzle became larger, and the duration of jet flow also increased, so that a larger droplet was formed after break-up. As an aside, the pressure in the nozzle cavity is difficult to balance in a short time when the duty ratio is small, which causes the droplet diameter to change significantly. However, the closing time of the nozzle should not be too short, otherwise it will lead to incomplete closing of the needle and nozzle, forming intermittent jets. The proposed dispenser has a wide dispensing range in terms of the size of droplets produced. Compared with some piezoelectric dispensers mentioned in the literature [3,25], our dispenser can obtain smaller droplets.

**Figure 13:**
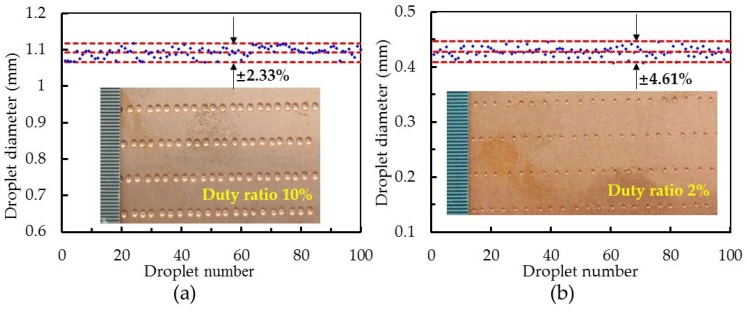
Variation in the droplet diameters for the jetting dispenser. (**a**) Duty ratio is 10% and (**b**) duty ratio is 2% (nozzle diameter: 200 µm; feed pressure: 3 bar; voltage amplitude: 144 V; frequency: 100 Hz; viscosity: 500 mPa∙s).

The consistency of droplets is an important index to evaluate the stability of a dispenser. In the experiment, 100 droplets at a square-wave duty ratio of 10% and 2% were randomly selected and their diameters measured. The results are shown in Figure 13. At a square-wave duty ratio of 10%, the average diameter of a droplet was 1.094 mm, and the variation range was ± 2.33%. At a square-wave duty ratio of 2%, the average diameter of a droplet was 0.428 mm, and the variation range was ± 4.61%. Compared with some jetting dispensers in the literature [26,27,28], our results indicate a steady performance of the proposed jetting dispenser for microscale droplet distribution. 

## 5. Conclusions

In this paper, the influence of needle structure on the jetting process of an on–off valve piezostack-driven dispenser was investigated through simulation and experimentation. (1) During the dispensing process, the needle with a side cap generated 8.27 MPa of pressure in the nozzle cavity, and produced droplets with an average volume of 1.166 μL. Respectively, the pressure and volume were 2.39 times and 0.94 times that produced by the model without a side cap. These results suggest that the needle with a side cap had an enhanced squeezing effect on the fluid compared to the needle without a side cap. This clearly increased the pressure of the nozzle cavity and the velocity of jet, and was also beneficial for obtaining small droplets. (2) For the needle with a side cap, the parameters—set at a pressure of 3 bar, a nozzle conic angle θ of 85°–105°, a needle conic angle α of 10°–35° and a side clearance β of 0.1–0.3 mm—made a dispenser with a large jet velocity and stable performance, which was able to produce microscale droplets. (3) A control method for the dispenser with an enhanced squeeze needle was proposed. The minimum droplet diameter obtained for this dispenser was 0.42 mm, measured at a duty cycle of 2%; the droplets variation range was ± 4.61%. 

The experimental results showed that the enhanced squeezing needle was able to obtain larger nozzle cavity pressure during the dispensing process, which is beneficial to the high frequency operation of the dispenser. By selecting appropriate structure parameters for the needle, the dispenser can obtain small droplets and have good droplet consistency. In future work, we will further study the dispensing process based on the fluid dynamics theory, and consider structural parameters—such as the nozzle and needle—in order to better guide the design and control of the dispenser. In addition, we will carry out dispensing experiments of fluids with different viscosities and verify the performance of the dispenser through more reasonable evaluation methods.

## Figures and Tables

**Table 1:** Initial values of each parameter in the fluid dynamics simulation.

**Parameter**	**Density of Adhesive**	**Dynamic Viscosity**	**Surface Tension Coefficient**	**Temperature of Adhesive**
Initial value	1263.31 kg/m^3^	500 mPa∙s	0.4 N/m	20 °C
**Parameter**	**Feed Pressure**	**Acceleration of Gravity**	**Needle Stroke**	**Velocity of Needle**
Initial value	0.3 MPa	9.8 m/s^2^	0.20 mm	Rising 0.3 m/s Falling 0.5 m/s

**Table 2:** Comparison of the droplet volume produced by the needles with and without side caps.

Needle Structure	Droplet Volume (μL)					
	First Cycle	Second Cycle	Third Cycle	Fourth Cycle	Fifth Cycle	Average
Needle with side cap	1.15	1.15	1.17	1.18	1.18	1.166
Needle without side cap	1.24	1.22	1.24	1.23	1.24	1.234
